# Supplementary material for: Possible intermediate quantum spin liquid phase in α-RuCl3 under high magnetic fields up to 100 T
Source: Nat Commun. 2023 Sep 12;14:5613. doi: 10.1038/s41467-023-41232-7 (PMC10497594; doi:10.1038/s41467-023-41232-7)
Supplement: Supplementary file 1 — Supplementary Information [file 41467_2023_41232_MOESM1_ESM.pdf]

Supplementary Materials for Possible Intermediate Quantum Spin Liquid Phase in α-RuCl$_3$ under High Magnetic Fields up to 100 T

Xu-Guang Zhou, Han Li, Yasuhiro H. Matsuda, Akira Matsuo, Wei Li, Nobuyuki Kurita, Gang Su, Koichi Kindo, and Hidekazu Tanaka

A. MORE EXPERIMENTAL DATA

In this section, we show the magnetization results for $\theta \simeq 90°$, i.e., under in-plane fields. As the in-plane magnetization has been intensively studied, the comparison below serves as a benchmark of the sample as well as the precision of our measurements. The M-T data for the sample after the single-turn coil pulse field experiments are also shown in this section, which is measured by SQUID.

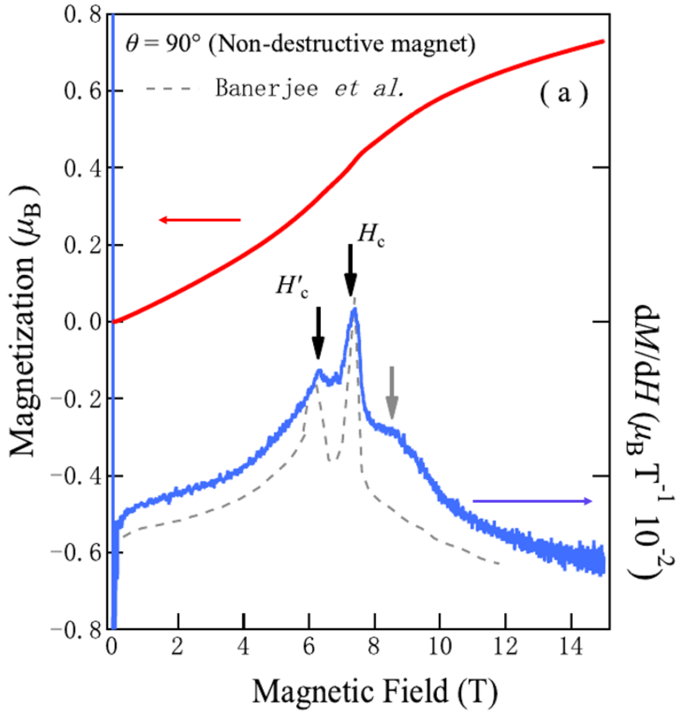

Supplementary Fig. 1. The magnetization curve and dM/dH data for $\theta = 90°$ measured by (a) non-destructive magnet. The grey dash line is the magnetic susceptibility results reported by Banerjee *et al.* [1] (which is ac susceptibility), in which two peak positions (indicated by the arrows) are in very good agreements with that of ours, except for the H_c^{AB} peak labelled by grey arrow.

In-plane field magnetization process. Supplementary Figure 1 shows the magnetization and dM/dH curves. In the dM/dH curve there exist three clear peaks at 6.2 T, 7.3 T, and 8.5 T, respectively, which are in excellent agreement with previous pulse-field measurements in Ref. [2]. We have also plotted the magnetic susceptibility data from Ref. [1], and find the 8.5 T peak marked with the grey arrow, which can be ascribed to the transition in the ABAB stacking fault component, which is absent in their sample. The peaks at 6.2 T and 7.3 T are marked as H_c' and H_c, respectively, which have been proposed to be the transition fields of two different zigzag antiferromagnetic phases [3, 4].

Sample condition after high pulse field. The sample qualities before and after the single-turn coil pulse field are also checked by measuring the M-T curves. In Fig. 1 of the main text, we show results of three independent

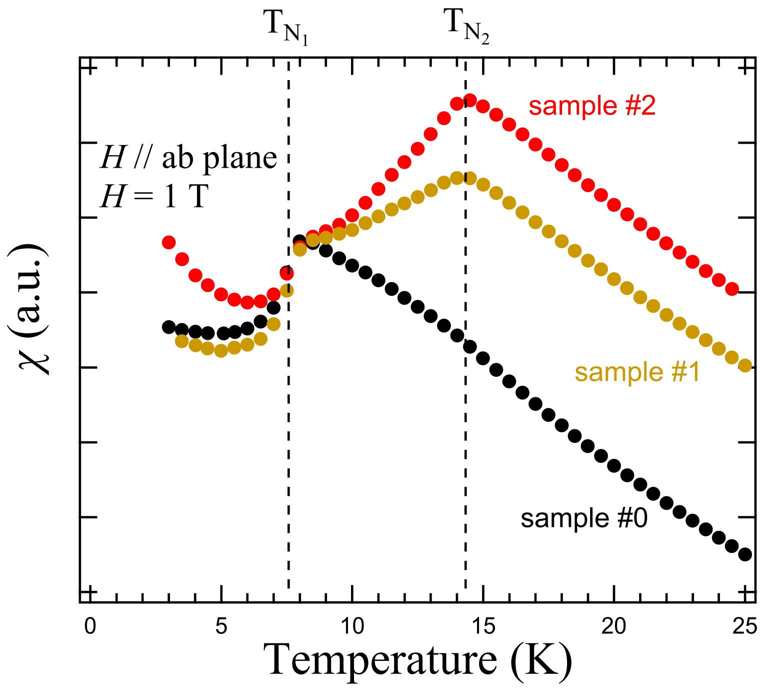

Supplementary Fig. 2. The M-T curves measured by SQUID. Sample #1 and #2 are measured after experiments (iii) and (i) (see Fig. 1 of the main text), respectively. Sample #0 has not been exposed to single-turn coil pulse field experiments.

single-turn coil pulse field experiments, namely case (i), (ii), and (iii). We have performed experiments (ii) and (iii) by employing the same sample (sample #1 in Supplementary Fig. 2), and case (i) by employing sample #2. In Supplementary Fig. 2, we have measured the M-T curves of the samples after the pulse field experiments (i) and (iii) [as the experiment (ii) is performed before (iii)]. As shown in Supplementary Fig. 2, for sample #1 (iii) and sample #2 (i) there are two features at 7 K and 14 K, which are ascribed to the onset of two zigzag order with ABC- and AB-type 3D stackings, respectively [5], in excellent agreement with previous observations in Refs. [2, 6]. For sample #0 (a sample has not been exposed to the single-turn coil pulse field experiment), the AB-type stacking rarely appears in the sample, as evidenced by the very weak 14 K signal [5]. In summary, the ABC stacking is still robust in the crystal after the pulse fields expereiments.

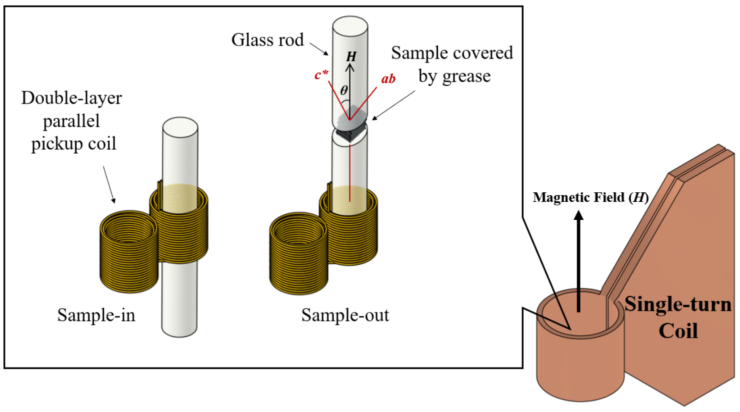

Supplementary Fig. 3. The schematic for the setup of sample, double-layer magnetization pickup coil, and the single-turn coil. The left panel in the zoomed-in segment shows the condition that sample is in the pick-up coil, while the right one shows the condition to measure the background of signals.

Setup of the single-turn coil magnetic field experiment and more data. Supplementary Fig. 3 shows the schematic for the setup of sample and double-layer magnetization pickup coil. When performing the single-turn coil experiment, the single-turn coil, pickup-coil, and the Kapton tube with sample are placed visually in parallel. The angle θ is determined by the inclined section the glass rod as shown in Supplementary Fig. 3. In order to ensure that no excess stress is applied to the sample while holding the sample between the two glass rods, vacuum silicone grease is employed to cover the sample. Because there are many degrees of freedom in this setup, the errorbar of angle θ between c* and the magnetic field is estimated to be 2.5°. Some other details of single-turn coil magnetic field experiment has been described in Ref. [7–9].

In Supplementary Fig. 4, we show the comparison between the up-sweep and down-sweep processes of dM/dH for experiment (i) and (iii). In the down-sweep process of experiment (i), we observed the signals at ∼8 T, ∼36 T, and ∼76 T, which correspond to the H_c^{AB}, H_c^l, and H_c^h, respectively. The signals show excellent consistency with those observed in the up-sweep process. In the down-sweep data of experiment (iii), we similarly observed the signals at ∼8 T, ∼35 T, and ∼83 T (the anomalous hump at 24 T may be ascribed to the transition from zigzag 1 to zigzag 2). Here, because the signals observed at the up-sweep and down-sweep field show consistency with each other, and there is no significant hysteresis between the up-sweep and down-sweep signals, we can exclude the possibility that the second phase transition is caused by the pealing out of sample or the structure phase transition of the sample, although there are some ABAB stacking fault component increases after our single-turn field experiment (see Supplementary Fig. 2). In addition, the phase transition at ∼8.5 T marked by H_c^{AB} is thought to be the feature of ABAB stacking fault component in α-$RuCl_3$, based on the Fig. 2 of our main text and the magnetization results in Ref. [2, 10].

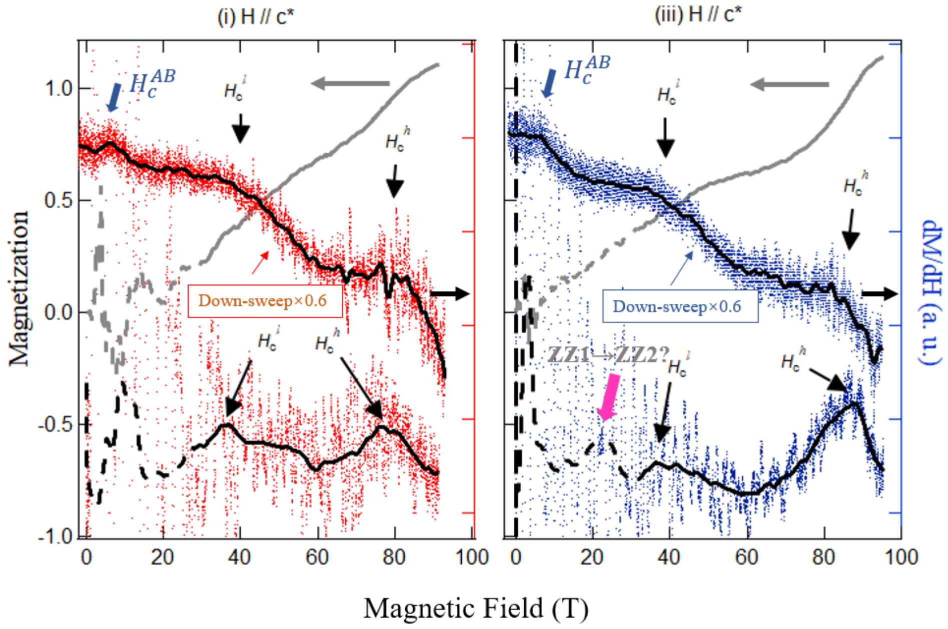

Supplementary Fig. 4. Comparison of magnetization processes between the up-sweep and down-sweep dM/dH data for experiment (i) and (iii). The gray curves represent the M-H processes. The dM/dH data are represented by the black curves. The anomaly marked by pink arrow may be ascribed to the phase transition from zigzag1 (ZZ1) to zigzag2 (ZZ2) which has been reported in Ref. [4]. The up-ward trend of dM/dH curves at low field during demagnetization is caused by the inharmonious of magnetic field during down-sweep process.

To further confirm that the transition signals H_c, H_c^l, and H_c^h are from the pristine ABC stacking components but not the ABAB stacking fault, we show the magnetization process and dM/dH data of a sample full of ABAB stacking fault under c*-axis external field up to 100 T. We damage α-$RuCl_3$ by deforming the sample and producing lots of ABAB stacking fault components, which now exhibits ordering temperature of about 14 K, as shown in Supplementary Fig. 5(a). The dM/dH curve and magnetization process are given in Supplementary Fig. 5(b), where the H_c^l (∼35 T) and H_c^h (∼80 T) transitions are absent in the ABAB stacking fault sample. This also evidences that the emergence of two phase transitions H_c^l and H_c^h in α-$RuCl_3$ for θ is in the vicinity of 0°, which support our conclusion of the intermediate QSL liquid phase between the transitions H_c^l and H_c^h.

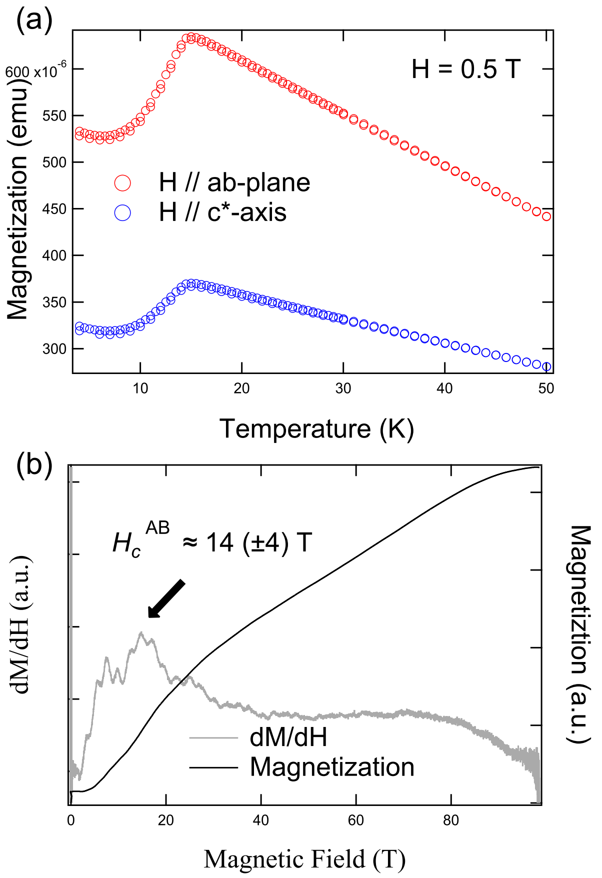

Supplementary Fig. 5. The magnetization curve and dM/dH data up to 100 T for a sample full of ABAB stacking fault components under the c*-axis external field. Only a 14 ±4 T peak is observed in the dM/dH curves.

B. DENSITY MATRIX RENORMALIZATION GROUP SIMULATIONS

In this section, we show the density matrix renormalization group (DMRG) calculations of the realistic K-J-Γ-Γ' model for α-$RuCl_3$ under fields applied along various θ angles.

The geometry adopted in DMRG simulations. The simulations are performed on the YC$W \times L \times 2$ geometry with W up to 6 and L up to 10, with kept bond dimension D up to 1024. The example of a YC4 $\times$ 6 $\times$ 2 lattice is illustrated in Supplementary Fig. 6(a), where x-, y-, and z-type bond also indicated in blue, green and red colors. An in-plane direction $[1\ 1\ \bar{2}]$ with $\theta = 90°$ is indicated by the red arrow in Supplementary Fig. 6(a).

The calculated dM/dH curves and transition fields. As shown in Supplementary Fig. 6(b,c), the quantum phase transition is clearly signatured by the divergent peaks in dM/dH. When $\theta < 3.5°$, two peaks at H_c^l and H_c^h can be clearly seen, which indicate the transition fields when the zigzag order gets suppressed (H_c^l) and the system enters the field-induced polarized phase (H_c^h), respectively. For clarity, all dM/dH curves are shifted vertically by about 0.1 μ_B/T, and the small θ data are seen to suffer relatively strong finite-size effects. As θ further increases, we find only a single transition field H_c from the zigzag-ordered phase to the polarized one. All the transition fields, namely, H_c^l, H_c^h and H_c are indicated by the arrows at the peak position of dM/dH curves.

The static spin-structure factors in various phases. In Supplementary Fig. 6(d-i), we show the contour plots of the spin structure factors in the Brillouin zone (BZ), for typical field angles $\theta = 0.8°$ and 1.4° and computed on YC4 geometries with $L = 6$ and 10. In particular, the z-component structure factors under the representative $H \simeq 32$ T,

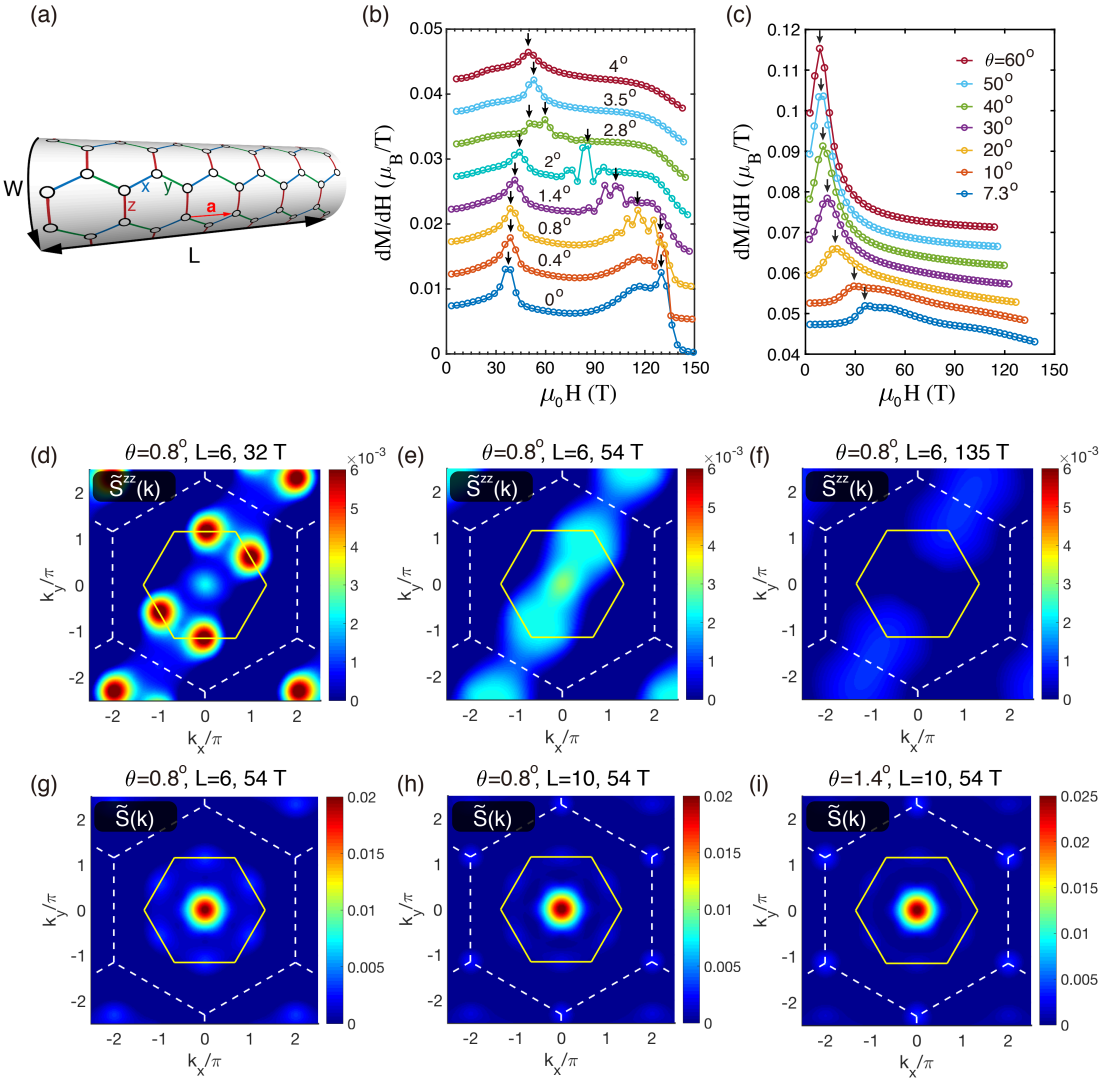

Supplementary Fig. 6. (a) The cylindrical geometry YC$W \times L \times 2$ employed in the DMRG simulations. (b,c) show the calculated dM/dH curves with small (b) and large (c) tilting angle θ. The estimated transition fields are indicated by the arrows. For a typical angle $\theta = 0.8°$, we show the contour plots of $\tilde{S}^{zz}(\mathbf{k})$ under (d) $H \simeq 32$ T, (e) $H \simeq 54$ T and (f) $H \simeq 135$ T on YC$4 \times L \times 2$ lattices (with $L = 6$), where the stripy background as well as the peculiar patterns for each phase can be clearly seen. (g, h) show the total structure factors $\tilde{S}(\mathbf{k})$ in the intermediate QSL phase, computed on cylinders of length $L = 6$ and 10, respectively, to check the convergence of the results. In panel (i), we show $\tilde{S}(\mathbf{k})$ with another small angle $\theta = 1.4°$, which resembles the results in panel (h) with $\theta = 0.8°$.

54 T and 135 T are shown, which is described by

$$\tilde{S}^{zz}(\mathbf{k}) = \frac{1}{N}\sum_j e^{i\mathbf{k}(\mathbf{r}_j - \mathbf{r}_{i_0})}(\langle S^z_{i_0} S^z_j\rangle - \langle S^z_{i_0}\rangle\langle S^z_j\rangle), \tag{1}$$

where N is the total site numbers, i_0 is a fixed central reference site, and j runs over the whole lattice.

In all three panels Supplementary Fig. 6(d-f) we see the stripy backgrounds that represent the short-range and bond-directional spin correlation due to the strong Kitaev term, as discussed in Ref. [11]. The bright M points in Supplementary Fig. 6(d) indicates the zigzag spin correlation in the ground state, which gets suppressed under fields $H \geq 35$ T and the spin structure becomes flattened and leaves only the stripy background in Supplementary Fig. 6(e). As field further increases, the system enters the spin-polarized phase with virtually no spin correlations (Eq. 1) remained.

Ground-state spin structures in the intermediate QSL phase. To further validate the intermediate QSL phase under small-angle fields, we show the contour plot of total spin structure factor $\tilde{S}(\mathbf{k}) = \sum_\gamma \tilde{S}^{\gamma\gamma}(\mathbf{k})$ with $\gamma = x, y, z$ in Supplementary Fig. 6(g-i) with $\theta = 0.8°$ and $1.4°$. There is no diverging peaks present in the structure factor and the brightness at the Γ point remains unchanged as the system size increases from $L = 6$ [Supplementary Fig. 6(g)] to $L = 10$ [Supplementary Fig. 6(h)]. The peak at M point that corresponds to the zigzag order gets fainter as L increases, indicating the absence of spontaneous long-range order in the intermediate phase. In Supplementary Fig. 6(i), we also provide the results with slightly larger $\theta = 1.4°$ which is very similar to Supplementary Fig. 6(h). These structure factor data indicate the absence of magnetic order in the intermediate-field regime and support the presence of a QSL phase in the field-angle quantum phase diagram illustrated in Fig. 4 of the main text.

Finite-size effect in DMRG calculations. As shown in Supplementary Fig. 7, in order to check the convergency of the calculated critical fields H_c^l and H_c^h in the finite-size simulations, we show the magnetizations and their derivatives on two geometries, i.e., YC4×6×2 and YC6×10×2 lattices, in Supplementary Fig. 7(a) and Supplementary Fig. 7(b), respectively. It can be seen that the finite-width effect has little influence on the determination of H_c^l and H_c^h for $\theta = 0°$. In addition, we note that the height of the low-field peak at H_c^l obtained by the larger lattice (YC6×10×2) is lower than that obtained on the small one (YC4×6×2), which seems to explain that the peaks of H_c^l obtained from the experiment is relatively weaker than the calculated one in Fig. 3 of the main text.

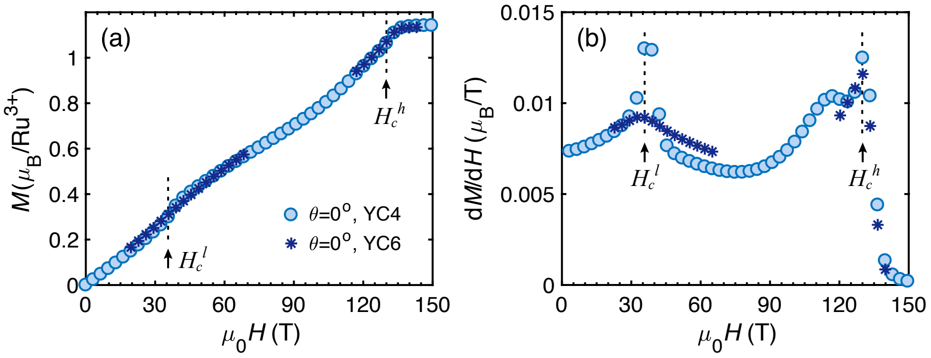

Supplementary Fig. 7. Comparison of magnetization curves obtained by two finite-size DMRG calculations. The asterisks represent the results simulated by YC6×10×2, where the solid circles represent the data calculated on YC4×6×2. The transition fields H_c^l and H_c^h are indicated by the black arrows, and are observed to be stable at around 35 T and 130 T for $\theta = 0°$, respectively.

[1] A. Banerjee, P. Lampen-Kelley, J. Knolle, C. Balz, A. A. Aczel, B. Winn, Y. Liu, D. Pajerowski, J. Yan, C. A. Bridges, A. T. Savici, B. C. Chakoumakos, M. D. Lumsden, D. A. Tennant, R. Moessner, D. G. Mandrus, and S. E. Nagler, Excitations in the field-induced quantum spin liquid state of α-$RuCl_3$, npj Quantum Materials **3**, 8 (2018).

[2] Y. Kubota, H. Tanaka, T. Ono, Y. Narumi, and K. Kindo, Successive magnetic phase transitions in α-$RuCl_3$: XY-like frustrated magnet on the honeycomb lattice, Phys. Rev. B **91**, 094422 (2015).

[3] C. Balz, P. Lampen-Kelley, A. Banerjee, J. Yan, Z. Lu, X. Hu, S. M. Yadav, Y. Takano, Y. Liu, D. A. Tennant, M. D. Lumsden, D. Mandrus, and S. E. Nagler, Finite field regime for a quantum spin liquid in α-$RuCl_3$, Phys. Rev. B **100**, 060405 (2019).

[4] C. Balz, L. Janssen, P. Lampen-Kelley, A. Banerjee, Y. H. Liu, J.-Q. Yan, D. G. Mandrus, M. Vojta, and S. E. Nagler, Field-induced intermediate ordered phase and anisotropic interlayer interactions in α-$RuCl_3$, Phys. Rev. B **103**, 174417 (2021).

[5] H. B. Cao, A. Banerjee, J.-Q. Yan, C. A. Bridges, M. D. Lumsden, D. G. Mandrus, D. A. Tennant, B. C. Chakoumakos, and S. E. Nagler, Low-temperature crystal and magnetic structure of α-$RuCl_3$, Phys. Rev. B **93**, 134423 (2016).

[6] A. Banerjee, C. A. Bridges, J. Q. Yan, A. A. Aczel, L. Li, M. B. Stone, G. E. Granroth, M. D. Lumsden, Y. Yiu, J. Knolle, S. Bhattacharjee, D. L. Kovrizhin, R. Moessner, D. A. Tennant, D. G. Mandrus, and S. E. Nagler, Proximate Kitaev quantum spin liquid behaviour in a honeycomb magnet, Nat. Mater. **15**, 733 (2016).

[7] S. Takeyama, R. Sakakura, Y. H. Matsuda, A. Miyata, and M. Tokunaga, Precise magnetization measurements by parallel self-compensated induction coils in a vertical single-turn coil up to 103 T, J. Phys. Soc. Jap. **81**, 014702 (2012).

[8] Y. H. Matsuda, N. Abe, S. Takeyama, H. Kageyama, P. Corboz, A. Honecker, S. R. Manmana, G. R. Foltin, K. P. Schmidt, and F. Mila, Magnetization of $SrCu_2(BO_3)_2$ in ultrahigh magnetic fields up to 118 T, Phys. Rev. Lett. **111**, 137204 (2013).

[9] X.-G. Zhou, Y. Yao, Y. H. Matsuda, A. Ikeda, A. Matsuo, K. Kindo, and H. Tanaka, Particle-hole symmetry breaking in a spin-dimer system $TlCuCl_3$ observed at 100 T, Phys. Rev. Lett. **125**, 267207 (2020).

[10] R. D. Johnson, S. C. Williams, A. A. Haghighirad, J. Singleton, V. Zapf, P. Manuel, I. I. Mazin, Y. Li, H. O. Jeschke, R. Valentí, and R. Coldea, Monoclinic crystal structure of α-$RuCl_3$ and the zigzag antiferromagnetic ground state, Phys. Rev. B **92**, 235119 (2015).

[11] H. Li, H.-K. Zhang, J. Wang, H.-Q. Wu, Y. Gao, D.-W. Qu, Z.-X. Liu, S.-S. Gong, and W. Li, Identification of magnetic interactions and high-field quantum spin liquid in α-$RuCl_3$, Nat. Commun. **12**, 4007 (2021).
